# Editorial: Attention and Methylphenidate

**DOI:** 10.3389/fnbeh.2020.00066

**Published:** 2020-04-28

**Authors:** Avi Avital, Iris Manor, David Coghill

**Affiliations:** ^1^Department of Neuroscience, Rappaport Faculty of Medicine, Emek Medical Center, Technion - Israel Institute of Technology, Haifa, Israel; ^2^ADHD Clinic, Geha MHC and Clalit, Sackler School of Medicine, Tel Aviv, Israel; ^3^Department of Paediatrics, Faculty of Medicine, Dentistry and Health Sciences, University of Melbourne, Melbourne, VIC, Australia; ^4^Department of Psychiatry, Faculty of Medicine, Dentistry and Health Sciences, University of Melbourne, Melbourne, VIC, Australia

**Keywords:** ADHD, diagnosis, methylphenidate, adherence, anxiety, dysregulation, dose, non-responder

Attention Deficit Hyperactivit Disorder, more simply known as ADHD, is a neurodevelopmental disorder characterized by inattention, hyperactivity, and impulsivity. There are three major presentations: primarily hyperactive and impulsive, primarily inattentive, and combined type (Gaub and Carlson, 1997). However, despite the differences between the subtypes, methylphenidate (MPH), a psychostimulant, is the primary pharmaceutical treatment of choice (Heal et al., 2009).

While MPH is largely an effective treatment across the subtypes (O'Driscoll et al., 2005), there is a large issue with adherence to treatment. In her contribution, Kritchman et al. findings suggested that acute MPH administration induces a delayed anxiogenic response. Considering ADHD-anxiety comorbidity, acute doses of MPH can induce anxiety, while, paradoxically, chronic MPH treatment has anxiolytic effects (Zubedat et al., 2019). There can be large difference in dose-response to MPH, even when a patient adheres to ADHD treatment. Additionally, some ADHD patients do not respond to medication at all, despite displaying typical ADHD symptoms. These “non-responders” may decrease adherence to MPH treatment, because they perceive the medication to be ineffective. The exact cause for non-responders is unknown, and it may stem from a puzzling, non-linear dose response to MPH.

The subset of ADHD non-responders may be mitigated with improved objective diagnostic assessments of ADHD. Throughout one's life, a variety of circumstances influence the individual's attention. For instance, high levels of stress, exhaustion and hunger, can all have large implications on attention, and are common dysregulating experiences throughout one's life. Since there are no sufficiently objective and accurate diagnostics for ADHD, subjects experiencing temporary inattention due to external circumstances may be misdiagnosed. These misdiagnoses could account for the number of non-responders to MPH treatment, as these “non-responders” could simply be patients suffering from transient inattention, rather than a true neurodevelopmental disorder. In the past 25 years the rates of ADHD diagnosis have increased (Mahone and Denckla, 2017), and while this may be due to a true increase in ADHD rates, lack of objective diagnostic protocols can increase misdiagnosis.

While these disparities in dose-response are puzzling, many techniques may shed light on these issues. In her contribution, Wigal et al. described the Laboratory School Protocol (LSP), a research protocol in which students are placed in a classroom environment where they are medicated and their behavior is evaluated. In her contribution, Rinker et al. postulated that ADHD affects academic functioning regardless of education level; Thus, LSPs are applicable to ADHD regardless of education level. In LSPs, medication is given at the research site to control for temporal differences in application, elucidating on the large variation in dose-response to MPH in ADHD patients. Wigal et al. reported that placebo effects are reduced when research environments mirror situations in which symptoms would be aggravated and when medication administration is tightly controlled, altogether aiding in the establishment of more objective ADHD diagnostic tests.

LSPs utilize the Permanent Product Measure of Performance Math Test (PERMP) as the most objective ADHD diagnostic test to date. However, promising research regarding norepinephrine receptors could lead to objective diagnosis even outside of simulated classroom environments like the LSP and exams like the PERMP. In their commentary on van den Brink et al. (2018), Medel et al. contributed that the differential expression of α1 and α2 norepinephrine (NE) receptors can induce heterogeneous spatial modes, a shifting of “brain states.” Thus, suggesting that there is a dynamic shaping of brain activity by neuromodulations through the heterogeneous expression of α1 and α2 NE receptors that creates spontaneous fluctuations in cognition, elucidating on data previously established by Shine et al. (2019). In her contributed mini-review, Wigal et al. further supports this suggestion by claiming that ADHD itself is not necessarily an attention deficit, but rather inconsistent and misdirected attention. Likewise, according to Spiegel and Pollak contributed research, ADHD encompasses a lack of proper evaluation of risk-taking behavior. Hypothetically, those with ADHD do not necessarily have limited attention, but the attention is misdirected through varying brain states due to differential expression of α1 and α2 NE receptors. Practically, Medel et al. show that pupillometry can measure varying levels of NE with different brain states, establishing a possible objective diagnostic measure of ADHD. Together, pupillometry and the PERPM may prove to be a robust, objective diagnostic measure of ADHD, which may limit misdiagnosis in ADHD and the number of “non-responders” to ADHD treatment.

While MPH and behavioral therapies are available, there are prevention techniques which may help to reduce the development of ADHD altogether. Tapping the black box of ADHD on its translational face, previous reports utilizing animal models showed that the application of MPH alone and/or with enriched environment (EE) on adult offspring rescues the detrimental effects of prenatal stress attentional reactivity (Zubedat et al., 2015). The influence of EE and MPH could be translated and confirmed by conducting LSP studies, hence, elucidating on ways EE may be increased in school systems to aid in ADHD symptoms.

Taken with these results, MPH has clinical applications outside of ADHD. Acute MPH improves cognitive ability in those with and without ADHD (Linssen et al., 2014). Temporary use of MPH to improve cognition may be appropriate in professions where inattention has true large implications in the livelihood of others (e.g., medical doctors on extended shift). However, as previously mentioned, side effects such as temporarily increased anxiety must be taken into account when MPH is acutely administered.

In essence, adherence issues with MPH treatment in those with ADHD may be rooted in acute increases in anxiety, non-responders, and misdiagnosis. These adherence issues could be mediated by assuring patients anxiogenic effects are temporary. In order to accurately diagnose patients with ADHD, we must focus on discovering and implementing objective diagnostic measures such as pupillometry. In order to decrease ADHD diagnosis overall, we can increase research on potential prevention methods such as environmental enrichment, as well as on the effects of adverse life events, such as prenatal stress, on attention.

## Author Contributions

AA conceptualized and wrote the article. IM and DC consulted and edited the paper.

## Conflict of Interest

Independent of this editorial DC has received research funding from Takeda/Shire, honoraria from Takeda/Shire, Medice and Servier. The remaining authors declare that the research was conducted in the absence of any commercial or financial relationships that could be construed as a potential conflict of interest.
